# Supplementary material for: A Quantitative Survey of Bacterial Persistence in the Presence of Antibiotics: Towards Antipersister Antimicrobial Discovery
Source: Antibiotics (Basel). 2020 Aug 13;9(8):508. doi: 10.3390/antibiotics9080508 (PMC7460088; doi:10.3390/antibiotics9080508)
Supplement: Supplementary file 1 [file antibiotics-09-00508-s001.pdf]

Table S1: Levels of bacteria persisting to antibiotics and antibiotic usage in the time-kill data. From left to right the data were grouped by growth phase, then the species of bacteria (full species names in main text, Table 1) followed by the descriptive statistics for persisters and the antibiotic usage. The statistics for persister levels were the median, mean and confidence interval 95% (CI95) as percentages of the cell count against time zero or against the control without antibiotics, if only one time point was reported. The statistics for antibiotic usage were the mode of the range for the minimal inhibitory concentration (MIC) - when reported, the mode for the range of concentrations of antibiotic used in the persister assays, and the mode of the total time of incubation (hours). The total number of time points collected for each set of species and antibiotics was taken here a sample size (n). All the data entries were matched to the 187 references selected for this work. The bibliographic details for these references appear below the end of the table in numerical order as well as in Materials and Methods in the main text. TSM: trimethoprim+sulphamethoxazole

**Growth phase**	**Species**	**Antibiotics**	**Persisters**			**Antibiotic usage**			n	**References**
			**Median %**	**Mean %**	**CI 95 %**	**MIC**	**Used**	**Incubation**		
Exponential	***E. coli***	Amikacin	4.74E-01	2.52E+01	[0 - 74.1]	1	6	0.25	4	[1]
		Amoxicillin	9.00E-01	9.00E-01	NA	NA	50	3	1	[2]
		Ampicillin	2.00E-02	4.61E+00	[2.33 - 6.88]	4	100	5	223	[1, 3–46]
		Carbenicillin	2.37E-02	1.80E-01	[0 - 0.43]	NA	150	4	12	[47, 48]
		Cefotaxime	1.00E-03	2.65E-03	[0 - 0.01]	NA	100	4	3	[49, 50]
		Cefoxitin	1.00E-02	1.00E-02	NA	NA	320	6	1	[5]
		Ceftazidime	2.00E-05	2.00E-05	NA	NA	50	5	1	[51]
		Ceftriaxone	2.49E+00	2.63E+01	[0 - 74.5]	0.05	0.3	0.25	4	[1]
		Ciprofloxacin	3.16E-02	6.36E+00	[2.22 - 10.5]	0.01	1	3	110	[1, 5, 6, 8, 9, 12–14, 17, 24, 28, 48, 49, 52–62]
		D-cycloserine	6.26E-01	1.92E+01	[0 - 44.8]	NA	50	0.25	8	[45]
		Gentamicin	2.51E-03	6.81E-01	[0 - 1.69]	4	7.5	5	32	[4–7, 24, 33]
		Kanamycin	1.79E-03	2.20E-02	[0 - 0.05]	NA	50	6	8	[21, 27, 28, 30]
		Meropenem	3.16E-01	1.69E+01	[0 - 49.5]	1	6	6	6	[1, 5, 63]
		Mitomycin C	1.00E-03	3.70E-03	[0 - 0.01]	NA	10	4	3	[49, 50]
		Moxifloxacin	5.50E+01	5.26E+01	[0 - 106]	NA	0.8	4	4	[64]
		Nalidixic acid	1.29E+00	2.09E+01	[6.74 - 35.1]	4	20	0.25	26	[65]
		Netilmicin	6.58E-04	1.68E+00	[0 - 4.76]	NA	2.8	3	10	[66, 67]
		Nitrofurantoin	6.70E+00	2.92E+01	[10.5 - 47.8]	12.5	12.5	0.25	20	[61]
		Norfloxacin	5.01E-02	5.86E+00	[0 - 13.2]	0.06	1	3	37	[7, 15, 21, 28, 31, 43, 62, 68]
		Ofloxacin	1.58E-02	4.94E+00	[0.78 - 9.1]	0.06	5	3	93	[4, 10, 23, 26–28, 30, 36, 38, 41, 50, 51, 62, 64, 65, 69–72]
		Oxacillin	3.16E-02	3.16E-02	NA	NA	2500	6	1	[5]
		Pefloxacin	5.01E-01	1.42E+01	[2.99 - 25.5]	0.06	0.3	0.25	33	[62, 65]
		Phosphomycin	5.01E-01	1.48E+01	[0 - 42.6]	NA	50	0.25	7	[45]
		Polymyxin B	5.68E+01	1.03E+02	[69.7 - 136]	1	1	0.5	21	[73]
		Rifampicin	6.31E+01	4.56E+01	[16.7 - 74.6]	NA	100	0.5	5	[17]
		Streptomycin	2.49E-04	2.49E-04	[0 - 0]	NA	25	4	2	[49]
		Tobramycin	3.01E-03	3.33E+01	[5.49 - 61.2]	NA	20	1	12	[41, 49, 50, 56, 74]
	S. aureus	Amikacin	6.31E-01	3.77E+01	[2.01 - 73.5]	NA	80	48	8	[75]
		Ampicillin	4.09E+01	4.12E+01	[22.4 - 60.0]	NA	100	3	4	[41]
		Carbenicillin	3.98E+00	3.98E+00	[3.98 - 3.98]	NA	500	3.5	3	[48]
		Cefotaxime	1.58E-03	2.51E-01	[0 - 0.57]	NA	60	48	8	[75]
		Ciprofloxacin	7.00E-02	4.93E+00	[1.06 - 8.8]	0.13	10	3	58	[9, 38, 75–82]
		Daptomycin	6.58E-04	1.09E-01	[0 - 0.27]	25	250	6	12	[81, 83]
		Erythromycin	2.24E+01	3.63E+01	[0 - 82.5]	NA	30	16	4	[84]
		Gentamicin	6.50E-02	3.20E+00	[0.61 - 5.78]	0.5	5	2	18	[77, 78, 82, 83]
		Linezolid	1.26E+01	1.28E+01	[9.51 - 16.1]	5	50	24	3	[83]
		Ofloxacin	6.31E-01	6.31E-01	NA	NA	5	2	1	[41]
		Oxacillin	3.98E-04	6.46E+00	[0 - 14.1]	0.13	30	48	18	[75, 78, 82, 84]
		Rifampicin	2.84E-01	7.41E+00	[0 - 15.9]	0.02	2	0.5	16	[17, 81, 83]
		Teicoplanin	7.94E+00	1.16E+01	[2.93 - 20.2]	1	100	8	15	[85]
		Tobramycin	1.26E-02	1.78E+00	[0.12 - 3.44]	0.6	60	1	26	[41, 74, 81, 86]
		TSM	2.00E+01	6.00E+01	[23.7 - 96.3]	2	200	4	10	[77]
		Vancomycin	1.00E+00	1.42E+01	[5.46 - 23.0]	0.5	50	48	31	[75, 78, 83, 85, 87]

Levels of bacteria persisting to antibiotics and antibiotic usage in the time-kill data (continued).

Growth phase	Species	Antibiotics	Persisters			Antibiotic usage			*n*	References
			Median %	Mean %	CI 95 %	MIC	Used	Incubation		
Exponential	***P. aeruginosa***	Amikacin	6.93E-05	5.40E-03	[0 - 0.02]	NA	40	1	6	[88]
		Carbenicillin	9.00E-03	1.90E-01	[0.01 - 0.36]	100	800	4	21	[48, 89–91]
		Ceftazidime	1.00E+02	8.18E+01	[62.5 - 101]	1.1	88	5	13	[92, 93]
		Ciprofloxacin	4.51E-02	5.80E-01	[0.13 - 1.02]	0.6	200	3.5	56	[9, 48, 53, 88, 93–98]
		Colistin	3.16E-06	3.16E-06	[0 - 0]	2	12	1	4	[89]
		Gentamicin	1.00E+01	1.00E+01	[10 - 10]	4	24	1	4	[89]
		Imipenem	7.51E-02	1.20E-01	[0.05 - 0.19]	4	320	24	12	[93]
		Levofloxacin	1.00E+01	8.00E+00	[4.08 - 11.9]	1	6	1	5	[89, 99]
		Meropenem	8.00E-05	8.00E-05	NA	NA	10	24	1	[100]
		Ofloxacin	2.84E-03	1.43E+00	[0 - 3.02]	NA	5	3	16	[38, 91]
		Polymyxin B	7.13E-02	1.63E-01	[0.05 - 0.28]	0.8	320	24	12	[93]
		Rifampicin	5.00E+00	5.00E+00	NA	NA	100	0.5	1	[17]
		Tobramycin	1.04E-02	4.49E+00	[0 - 10.3]	1.3	104	24	12	[93]
	A. baumannii	Amikacin	3.50E-01	3.25E-01	[0.16 - 0.49]	2	80	3	4	[101, 102]
		Ceftazidime	1.26E-01	2.42E-01	[0 - 0.5]	4	200	20	7	[103]
		Ciprofloxacin	1.00E-02	1.02E+00	[0 - 2.97]	0.5	25	2	8	[102, 103]
		Colistin	2.00E-01	1.74E+00	[0 - 4.16]	2	20	24	8	[101, 102, 104]
		Imipenem	6.99E-02	1.79E+00	[0 - 4.21]	4	200	20	8	[102, 103]
		Meropenem	1.00E-01	5.15E-01	[0.07 - 0.96]	1	15	48	11	[105, 106]
		Polymyxin B	1.26E-05	3.70E-03	[0 - 0.01]	2	100	4	7	[103]
		Rifampicin	5.00E+00	4.64E+00	[1.74 - 7.54]	4	80	3	3	[101]
		Tetracycline	3.16E-02	3.16E-02	[0.03 - 0.03]	1	50	1	7	[103]
		Tobramycin	6.58E-04	4.11E-03	[0 - 0.01]	4	200	4	12	[103, 105]
	M. tuberculosis	Ciprofloxacin	3.76E-01	1.06E+01	[0.89 - 20.3]	0.63	31.3	72	14	[107]
		D-cycloserine	5.25E-02	1.05E+02	[0 - 248]	NA	100	72	14	[107]
		Ethambutol	8.16E+00	1.02E+01	[1.23 - 19.1]	5	102	504	6	[108, 109]
		Isoniazid	1.00E+00	2.80E+00	[0.79 - 4.8]	0.14	1	168	20	[107–109]
		Rifampicin	3.16E-01	1.41E+01	[1.93 - 26.3]	0.1	1	168	23	[107–111]
		Streptomycin	7.94E-01	1.24E+01	[3.01 - 21.8]	0.63	31.25	72	18	[107, 110]
	S. aureus MRSA	Chloramphenicol	7.94E+00	9.58E+00	[3.27 - 15.9]	50	500	72	3	[112]
		Ciprofloxacin	6.31E-02	1.47E-01	[0 - 0.31]	5	50	48	3	[112]
		Clindamycin	3.16E-02	5.41E-01	[0 - 1.56]	500	5000	72	3	[112]
		Gentamicin	6.31E-02	1.35E+00	[0 - 3.93]	20	200	72	3	[112]
		Rifampicin	3.16E+02	4.65E+02	[0 - 1006]	0.4	4	24	3	[112]
		Tetracycline	1.26E+01	3.23E+01	[0 - 78.7]	50	500	72	3	[112]
		Vancomycin	1.00E+00	2.01E+01	[0 - 43.6]	1	10	24	11	[87, 112, 113]
	S. enterica	Ampicillin	1.26E-02	1.52E-01	[0 - 0.41]	NA	100	2	15	[41, 114, 115]
		Cefotaxime	1.32E-01	7.37E-01	[0 - 1.94]	12.5	100	4	8	[116–118]
		Ceftazidime	1.00E+01	2.71E+01	[0 - 63.4]	1.25	125	0.25	5	[119]
		Ciprofloxacin	2.51E-02	4.06E+00	[0 - 11.9]	0.03	0.03	2	25	[114, 115, 117, 119, 120]
		Gentamicin	1.00E-04	2.00E+01	[0 - 59.2]	12.5	100	2	5	[117, 121]
		Ofloxacin	3.16E-04	3.16E-04	[NA - NA]	NA	5	2	1	[41]
		Polymyxin B	2.84E-02	3.90E-02	[0.01 - 0.07]	NA	5	1	4	[115]
		Tobramycin	5.01E-04	5.01E-04	NA	NA	50	2	1	[41]
	L. monocytogenes	Ampicillin	1.58E+01	3.66E+01	[2.01 - 71.1]	3.13	185	72	5	[122]
		Erythromycin	6.31E+01	7.05E+01	[56.0 - 84.9]	0.2	6	10	5	[122]
		Gentamicin	5.50E-02	1.83E+02	[0 - 505]	NA	10	4	6	[123]
		Norfloxacin	3.57E-03	1.92E+00	[0 - 4.58]	3.13	100	4	16	[122, 123]
		TSM	1.58E-01	2.02E+01	[0 - 59.3]	0.78	95	72	5	[122]
		Vancomycin	6.31E+00	2.99E+01	[0 - 63.4]	1.56	47	72	5	[122]
	S. epidermidis	Ampicillin	5.01E-02	5.01E-02	NA	NA	100	2	1	[41]
		Ciprofloxacin	2.00E+00	2.00E+00	NA	NA	20	3	1	[9]
		Levofloxacin	7.94E-03	3.43E-02	[0 - 0.08]	0.06	25	3	19	[124]
		Ofloxacin	6.31E+01	6.31E+01	NA	NA	5	2	1	[41]
		Tobramycin	1.00E-03	1.00E-03	NA	NA	50	2	1	[41]
		Vancomycin	1.00E-01	1.37E+01	[0 - 39.5]	2	50	16	19	[124]
	S. mutans	Cefotaxime	1.00E+01	1.68E+01	[0 - 40.0]	NA	2	24	3	[125]
		Mitomycin C	6.70E-03	6.70E-03	NA	NA	0.5	24	1	[126]
		Ofloxacin	3.71E-01	3.34E+00	[0 - 9.39]	NA	20	24	4	[125, 126]
		Oxacillin	6.10E+00	1.30E+01	[0 - 31.0]	NA	2	24	4	[125, 126]
		Rifampicin	1.00E+01	1.70E+01	[0 - 39.9]	NA	50	24	3	[125]
		Vancomycin	1.00E+01	1.83E+01	[0 - 39.6]	NA	20	24	3	[125]
	S. suis	Amoxicillin	2.51E+00	4.00E+00	[0.42 - 7.58]	6.25	625	8	5	[127]
		Ciprofloxacin	1.00E+00	3.49E+00	[0 - 7.41]	1.56	156	8	5	[127]
		Daptomycin	1.26E-05	3.01E-05	[0 - 0]	3.12	312	2	5	[127]
		Gentamicin	7.94E-03	8.17E-03	[0.01 - 0.01]	25	2500	8	5	[127]
		Penicillin	2.51E+00	4.00E+00	[0.42 - 7.58]	0.05	5	8	5	[127]
		Rifampicin	5.01E+01	5.28E+01	[36.2 - 69.4]	3.12	312	8	5	[127]
	B. burgdorferi	Amoxicillin	2.00E-02	9.06E-01	[0 - 2.61]	NA	6	120	11	[2, 128]
		Ceftriaxone	1.26E-02	5.66E-01	[0 - 1.63]	NA	3	120	11	[2, 128]
		Doxycycline	6.31E-01	1.34E+00	[0 - 2.67]	0.25	5	120	9	[2, 128–130]
		Metronidazole	4.80E+01	4.80E+01	NA	25	8.55	96	1	[130]
		Penicillin	2.50E+01	2.50E+01	NA	NA	12.16	96	1	[130]
	K. pneumoniae	Ceftriaxone	2.00E-01	7.01E-01	[0 - 1.63]	0.05	5	24	5	[131]
		Ciprofloxacin	1.26E-02	1.60E+00	[0 - 4.7]	0.02	0.25	24	5	[131]
		Kanamycin	6.31E-05	4.78E-05	[0 - 0]	1.5	50	24	3	[131]

Levels of bacteria persisting to antibiotics and antibiotic usage in the time-kill data (continued).

Growth phase	Species	Antibiotics	Persisters			Antibiotic usage			*n*	References
			Median %	Mean %	CI 95 %	MIC	Used	Incubation		
Exponential	***B. subtilis***	Ampicillin	1.00E+02	1.00E+02	NA	NA	100	2	1	[41]
		Ofloxacin	7.94E-02	7.94E-02	NA	NA	5	2	1	[41]
		Tobramycin	1.58E-03	1.58E-03	NA	NA	50	2	1	[41]
	B. thuringiensis	Ampicillin	1.00E+01	1.00E+01	NA	NA	100	2	1	[41]
		Ofloxacin	1.00E-01	1.00E-01	NA	NA	5	2	1	[41]
		Tobramycin	7.94E-02	7.94E-02	NA	NA	50	2	1	[41]
	C. difficile	Cefalexin	6.31E-02	3.36E+00	[0 - 9.87]	5	50	144	3	[132]
		Pefloxacin	1.00E-01	9.65E-02	[0.02 - 0.17]	2.5	6.25	144	3	[132]
		Vancomycin	3.16E-01	5.44E-01	[0.1 - 0.99]	0	0.02	96	3	[132]
	S. flexneri	Ampicillin	6.31E-02	6.31E-02	NA	NA	100	2	1	[41]
		Ofloxacin	7.94E-04	7.94E-04	NA	NA	5	2	1	[41]
		Tobramycin	1.26E-02	1.26E-02	NA	NA	50	2	1	[41]
	S. pneumoniae	Levofloxacin	1.11E+01	2.90E+01	[6.63 - 51.4]	1	6	0.25	10	[133]
		Linezolid	4.74E-01	1.66E+01	[0 - 36.1]	1	18	0.25	10	[133]
		Vancomycin	4.74E-01	1.99E+01	[2.56 - 37.2]	0.5	30	8	18	[133, 134]
	S. saprophyticus	Ampicillin	1.00E-03	1.00E-03	NA	0.13	100	3	1	[24]
		Ciprofloxacin	1.70E-01	1.70E-01	NA	0.25	5	3	1	[24]
		Gentamicin	3.00E-03	3.00E-03	NA	0.06	10	3	1	[24]
	B. abortus	Ampicillin	2.00E-03	5.31E-02	[0 - 0.13]	2	100	1	5	[135]
		Gentamicin	2.51E-01	7.88E-01	[0 - 1.69]	0.67	40	1	5	[135]
	B. melitensis	Ampicillin	5.01E-02	2.55E-01	[0 - 0.63]	2	100	1	5	[135]
		Gentamicin	1.00E-03	6.29E-03	[0 - 0.02]	0.67	40	1	5	[135]
	B. pseudomallei	Ciprofloxacin	4.05E-02	4.70E-02	[0.01 - 0.09]	2	200	24	6	[136]
		Levofloxacin	1.10E-01	1.10E-01	NA	4	40	16	1	[99]
	V. vulnificus	Lomefloxacin	2.84E-01	1.04E+01	[0 - 29.9]	0.12	0.12	0.25	10	[137]
		Moxifloxacin	1.00E-02	1.00E+01	[0 - 29.6]	0.06	0.12	0.25	10	[137]
	B. thailandensis	Levofloxacin	1.40E-01	1.40E-01	NA	4	40	16	1	[99]
	B. cenocepacia	Levofloxacin	5.00E-02	8.67E-02	[0 - 0.19]	4	40	16	3	[99]
	B. multivorans	Levofloxacin	2.55E-01	2.74E-01	[0.11 - 0.44]	16	160	16	4	[99]
	L. lactis	Ampicillin	1.78E+00	7.90E+01	[0 - 217]	NA	100	0.25	7	[138]
	M. bovis	Isoniazid	7.13E+00	5.97E+00	[3.7 - 8.24]	NA	6.86	24	8	[139]
	P. mirabilis	Ampicillin	5.00E+01	5.17E+01	[21.8 - 81.5]	NA	50	0.5	8	[42]
	P. putida	Ciprofloxacin	7.00E-04	7.00E-04	[0 - 0]	NA	1.25	4	2	[140]
	S. marcescens	Ciprofloxacin	3.00E-03	3.00E-03	NA	NA	20	3	1	[9]
	S. sonnei	Ciprofloxacin	1.00E-01	2.01E+01	[0 - 59.3]	0.008	0.032	0.25	5	[141]
	V. cholerae	Gentamicin	1.53E+00	8.28E+00	[0 - 22.5]	NA	100	1.5	4	[142]

Levels of bacteria persisting to antibiotics and antibiotic usage in the time-kill data (continued).

Growth phase	Species	Antibiotics	Persisters			Antibiotic usage			n	References
			Median %	Mean %	CI 95 %	MIC	Used	Incubation		
Stationary	***E. coli***	Amikacin	1.50E-01	2.75E-01	[0 - 0.56]	NA	100	1	4	[143]
		Amoxicillin	1.00E+00	6.67E-01	[0.01 - 1.32]	NA	25	24	3	[144]
		Ampicillin	2.00E+00	3.10E+01	[20.9 - 41.1]	2	100	3	68	[6–8, 24, 33, 144–149]
		Carbenicillin	4.00E-01	4.00E-01	[0.29 - 0.51]	NA	500	3.5	3	[48]
		Cefepime	1.04E-03	1.67E+01	[0 - 49.3]	0.03	0.3	0	6	[150]
		Cefotaxime	5.43E-03	1.67E+01	[0 - 49.3]	0.06	0.6	0	6	[150]
		Cefpirome	1.10E-03	1.67E+01	[0 - 49.3]	0.06	0.6	0	6	[150]
		Chloramphenicol	1.65E+01	2.47E+01	[1.06 - 48.3]	NA	50	24	6	[144]
		Ciprofloxacin	6.16E+00	1.39E+01	[9.02 - 18.9]	0.05	5	5	64	[6, 8, 20, 24, 48, 53, 54, 59, 151, 152]
		Gentamicin	6.00E+00	2.17E+01	[11.8 - 31.6]	8	20	5	47	[6, 7, 24, 33, 145, 153–156]
		Kanamycin	1.00E+00	3.13E+00	[1.31 - 4.95]	NA	60	24	13	[33, 156]
		Netilmicin	6.31E+01	6.76E+01	[33.28 - 101]	NA	2.8	3	3	[67]
		Norfloxacin	2.51E+00	8.73E+00	[0.58 - 16.9]	0.13	5	24	19	[7, 21, 144, 155]
		Ofloxacin	9.00E-01	3.57E+00	[1.91 - 5.23]	0.05	5	5	56	[19, 51, 70, 72, 143–145, 149, 157–162]
		Rifampicin	9.00E+01	6.32E+01	[22.8 - 103]	6.25	100	24	5	[17, 145]
		Streptomycin	1.00E+00	1.00E+00	[1 - 1]	NA	75	24	3	[144]
		Tetracycline	3.25E+01	4.42E+01	[15.0 - 73.4]	1.2	50	24	12	[144, 145, 155]
		Tobramycin	1.00E+00	1.00E+00	NA	NA	100	5	1	[51]
		Trimethoprim	1.00E-01	2.34E+00	[0 - 6.14]	NA	64	24	5	[155]
	P. aeruginosa	Amikacin	3.16E-04	8.50E-04	[0 - 0]	1	400	5	12	[92, 163]
		Azithromycin	7.94E+00	8.92E+01	[0 - 198]	64	256	0.167	7	[164]
		Aztreonam	5.01E-01	1.11E+02	[0 - 283]	64	256	0.167	7	[164]
		Carbenicillin	5.00E+01	6.00E+01	[6.51 - 113]	NA	500	3.5	6	[48, 91]
		Ceftazidime	5.01E+01	6.26E+01	[49.8 - 75.3]	1.1	5.5	1	12	[93]
		Ciprofloxacin	1.26E-01	2.32E-01	[0.05 - 0.41]	0.6	200	1	23	[48, 53, 93]
		Clathromycin	1.58E+00	9.41E+00	[0 - 20.1]	64	256	0.167	7	[164]
		Colistin	2.51E-04	1.44E+00	[0 - 3.25]	64	256	0.167	7	[164]
		Gentamicin	1.20E-02	8.54E-01	[0 - 2.48]	4	2000	0.5	6	[154]
		Imipenem	3.16E-01	3.66E+00	[1.14 - 6.19]	4	20	1	12	[93]
		Meropenem	1.58E+01	1.58E+01	[0 - 46.8]	0.5	5	24	2	[94, 165]
		Ofloxacin	3.16E-01	7.36E+00	[0 - 18.8]	1	10	6	17	[91, 92, 94, 166–169]
		Polymyxin B	6.31E-01	3.65E+00	[1.2 - 6.1]	0.8	20	1	12	[93]
		Tobramycin	1.54E+01	7.22E+01	[23.4 - 120]	1.3	16	24	26	[91, 93, 94, 164, 170]
	S. aureus	Amikacin	1.00E+02	1.00E+02	[100 - 100]	32	10	5	7	[163]
		Carbenicillin	2.00E+00	2.00E+00	[2 - 2]	NA	500	3.5	3	[48]
		Ciprofloxacin	6.31E+00	8.23E+00	[1.17 - 15.3]	0.5	20	24	9	[78, 171–173]
		Daptomycin	3.16E-02	3.10E+01	[15.7 - 46.2]	25	400	3	33	[81, 83, 174–176]
		Erythromycin	1.14E+02	1.14E+02	[55.7 - 171]	NA	30	16	2	[84]
		Flucloxacillin	5.00E-01	5.00E-01	NA	0.25	10	24	1	[173]
		Gentamicin	2.51E+00	9.56E+00	[4.38 - 14.7]	2	400	5	32	[83, 154, 163, 177, 178]
		Levofloxacin	2.49E+00	8.95E+00	[2.88 - 15.0]	0.25	50	24	18	[172, 178, 179]
		Linezolid	1.00E+01	1.34E+01	[4.83 - 22.0]	5	33.73	24	9	[83, 176]
		Oxacillin	3.00E+01	3.72E+01	[2.73 - 71.6]	0.25	25	16	6	[84, 171]
		Rifampicin	4.16E+00	8.18E+00	[2.32 - 14.0]	0.06	2	24	12	[78, 83, 177, 178]
		Tobramycin	1.00E+02	1.00E+02	[100 - 100]	0.6	60	1	8	[81]
		Vancomycin	2.00E+01	4.85E+01	[27.5 - 69.6]	2	25	24	15	[83, 87, 176, 178]
	M. tuberculosis	Ciprofloxacin	6.31E+01	6.31E+01	[36.6 - 89.6]	0.5	5	120	12	[107, 180]
		D-cycloserine	3.16E+00	1.34E+02	[0 - 392]	NA	100	120	3	[107]
		Gatifloxacin	1.79E+01	2.73E+01	[4.52 - 50.0]	0.125	0	120	8	[180]
		Levofloxacin	4.50E+01	4.82E+01	[27.1 - 69.3]	0.354	0	120	8	[180]
		Moxifloxacin	1.79E+01	2.73E+01	[4.52 - 50.0]	0.177	0	120	8	[180]
		Ofloxacin	6.70E+01	5.86E+01	[35.4 - 81.7]	0.71	0	120	8	[180]
		Rifampicin	1.00E-02	2.44E-02	[0 - 0.06]	NA	64	72	3	[111]
		Streptomycin	6.00E-01	1.30E+00	[0 - 3.1]	0.63	40	120	4	[107]
	S. suis	Amoxicillin	5.01E+01	5.89E+01	[32.2 - 85.5]	6.25	625	1	5	[127]
		Ciprofloxacin	1.00E+01	3.41E+01	[0 - 70.4]	1.56	156	1	5	[127]
		Daptomycin	1.58E-04	1.58E-04	[0 - 0]	3.12	312	1	5	[127]
		Gentamicin	5.01E+01	5.21E+01	[24.1 - 80.1]	25	2500	1	5	[127]
		Penicillin	5.01E+00	2.51E+01	[0 - 62.1]	0.05	5	1	5	[127]
		Rifampicin	1.00E+02	1.00E+02	[100 - 100]	3.12	312	1	5	[127]
	L. monocytogenes	Ampicillin	1.00E+02	9.08E+01	[72.7 - 108]	1	3	6	4	[181]
		Gentamicin	1.16E-03	2.16E-03	[0 - 0.01]	2.5	10	6	4	[181]
		Nitrofurantoin	3.18E+00	3.44E+01	[0 - 75.1]	NA	400	0	6	[123]
		Norfloxacin	3.16E-02	1.06E+01	[0 - 27]	25	100	6	12	[123, 181]
		TSM	1.00E+02	7.75E+01	[33.4 - 121]	2.5	10	6	4	[181]
	A. baumannii	Amikacin	2.00E-04	1.87E-03	[0 - 0]	4	400	5	15	[163]
		Ciprofloxacin	1.08E+00	4.71E+00	[0 - 10.0]	0.5	500	1	6	[182]
		Colistin	7.94E-04	1.61E-01	[0 - 0.47]	0.5	500	1	5	[182]
		Meropenem	2.51E-03	2.51E-03	NA	NA	10	24	1	[165]

Levels of bacteria persisting to antibiotics and antibiotic usage in the time-kill data (continued).

Growth phase	Species	Antibiotics	Persisters			Antibiotic usage			*n*	References
			Median %	Mean %	CI 95 %	MIC	Used	Incubation		
Stationary	***S. aureus MRSA***	Ciprofloxacin	1.00E+02	1.00E+02	NA	0.5	5	8	1	[183]
		Gentamicin	1.26E+02	1.97E+02	[81.7 - 312]	1	10	8	7	[183–185]
		Levofloxacin	2.00E+00	1.70E+01	[0 - 40.8]	0.5	50	24	5	[179]
		Vancomycin	1.58E+01	3.21E+01	[4.93 - 59.2]	1	10	8	9	[87, 183, 184]
	S. epidermidis	Levofloxacin	2.05E+01	2.67E+01	[9.12 - 44.3]	0.06	25	7	6	[124]
		Vancomycin	2.05E+01	2.70E+01	[9.78 - 44.3]	2	50	7	6	[124]
	B. burgdorferi	Amoxicillin	1.50E+00	9.34E+00	[0 - 19.7]	NA	6	120	6	[2, 128]
		Ceftriaxone	8.29E-01	7.13E+00	[0 - 15.7]	NA	3	120	6	[2, 128]
		Doxycycline	5.63E-01	1.73E+01	[0 - 38.5]	0.25	10	120	8	[2, 129, 130]
		Metronidazole	9.40E+01	9.40E+01	NA	25	8.55	96	1	[130]
		Penicillin	8.40E+01	8.40E+01	NA	NA	12.16	96	1	[130]
	K. pneumoniae	Amikacin	1.00E-02	4.06E-01	[0 - 1.16]	1	400	5	13	[163]
		Meropenem	1.00E-01	1.00E-01	NA	0.5	5	2	1	[186]
	S. saprophyticus	Ampicillin	9.00E+01	9.00E+01	NA	0.13	100	3	1	[24]
		Ciprofloxacin	9.00E+01	9.00E+01	NA	0.25	5	3	1	[24]
		Gentamicin	7.00E+01	7.00E+01	NA	0.06	10	3	1	[24]
	B. thailandensis	Meropenem	6.31E-04	6.31E-04	NA	NA	10	24	1	[165]
	E. faecium	Amikacin	1.00E+02	1.00E+02	[100 - 100]	8	10	5	7	[163]
		Gentamicin	2.51E+01	4.25E+01	[20.4 - 64.6]	8	400	5	15	[163]
	E. aerogenes	Amikacin	2.00E-01	5.52E-01	[0 - 1.36]	4	400	5	15	[163]
	L. lactis	Ampicillin	5.66E+00	2.04E+01	[0 - 43.8]	NA	100	0	8	[138]
	M. smegmatis	Norfloxacin	1.50E+01	1.50E+01	[0 - 44.4]	2	20	24	2	[187]
	S. enterica	Ciprofloxacin	7.94E-03	2.44E-02	[0 - 0.06]	0.03	0.12	1	5	[120]

References

1. Debbia, E. A., Roveta, S., Schito, A. M., Gualco, L. & Marchese, A. Antibiotic persistence: the role of spontaneous DNA repair response. *Microb Drug Resist* **7,** 335–42. ISSN: 1076-6294 (Print) 1076-6294 (Linking). https://www.ncbi.nlm.nih.gov/pubmed/11822773 (2001).

2. Feng, J., Auwaerter, P. G. & Zhang, Y. Drug combinations against Borrelia burgdorferi persisters in vitro: eradication achieved by using daptomycin, cefoperazone and doxycycline. *PLoS One* **10,** e0117207. ISSN: 1932-6203 (Electronic) 1932-6203 (Linking). https://www.ncbi.nlm.nih.gov/pubmed/25806811 (2015).

3. Kim, J. S., Chowdhury, N., Yamasaki, R. & Wood, T. K. Viable but non-culturable and persistence describe the same bacterial stress state. *Environ Microbiol* **20,** 2038–2048. ISSN: 1462-2920 (Electronic) 1462-2912 (Linking). https://www.ncbi.nlm.nih.gov/pubmed/29457686 (2018).

4. Yamamoto, N. *et al.* Stochastic expression of lactate dehydrogenase A induces Escherichia coli persister formation. *J Biosci Bioeng* **126,** 30–37. ISSN: 1347-4421 (Electronic) 1347-4421 (Linking). https://www.ncbi.nlm.nih.gov/pubmed/29449156 (2018).

5. Molina-Quiroz, R. C. *et al.* Cyclic AMP Regulates Bacterial Persistence through Repression of the Oxidative Stress Response and SOS-Dependent DNA Repair in Uropathogenic Escherichia coli. *mBio* **9.** ISSN: 2150-7511 (Electronic). https://www.ncbi.nlm.nih.gov/pubmed/29317513 (2018).

6. Harms, A., Fino, C., Sorensen, M. A., Semsey, S. & Gerdes, K. Prophages and Growth Dynamics Confound Experimental Results with Antibiotic-Tolerant Persister Cells. *mBio* **8.** ISSN: 2150-7511 (Electronic). https://www.ncbi.nlm.nih.gov/pubmed/29233898 (2017).

7. Liu, S. *et al.* Variable Persister Gene Interactions with (p)ppGpp for Persister Formation in Escherichia coli. *Front Microbiol* **8,** 1795. ISSN: 1664-302X (Print) 1664-302X (Linking). https://www.ncbi.nlm.nih.gov/pubmed/28979246 (2017).

8. Shen, Q. *et al.* Bactericidal activity of alpha-bromocinnamaldehyde against persisters in Escherichia coli. *PLoS One* **12,** e0182122. ISSN: 1932-6203 (Electronic) 1932-6203 (Linking). https://www.ncbi.nlm.nih.gov/pubmed/28750057 (2017).

9. Wang, M. *et al.* Medium chain unsaturated fatty acid ethyl esters inhibit persister formation of Escherichia coli via antitoxin HipB. *Appl Microbiol Biotechnol* **102,** 8511–8524. ISSN: 1432-0614 (Electronic) 0175-7598 (Linking). https://www.ncbi.nlm.nih.gov/pubmed/30088019 (2018).

10. Goormaghtigh, F. *et al.* Reassessing the Role of Type II Toxin-Antitoxin Systems in Formation of Escherichia coli Type II Persister Cells. *mBio* **9.** ISSN: 2150-7511 (Electronic). https://www.ncbi.nlm.nih.gov/pubmed/29895634 (2018).

11. Kim, J. S., Yamasaki, R., Song, S., Zhang, W. & Wood, T. K. Single cell observations show persister cells wake based on ribosome content. *Environ Microbiol* **20,** 2085–2098. ISSN: 1462-2920 (Electronic) 1462-2912 (Linking). https://www.ncbi.nlm.nih.gov/pubmed/29528544 (2018).

12. Wang, T., El Meouche, I. & Dunlop, M. J. Bacterial persistence induced by salicylate via reactive oxygen species. *Sci Rep* **7,** 43839. ISSN: 2045-2322 (Electronic) 2045-2322 (Linking). https://www.ncbi.nlm.nih.gov/pubmed/28281556 (2017).

13. Guo, Y. *et al.* Tail-Anchored Inner Membrane Protein ElaB Increases Resistance to Stress While Reducing Persistence in Escherichia coli. *J Bacteriol* **199.** ISSN: 1098-5530 (Electronic) 0021-9193 (Linking). https://www.ncbi.nlm.nih.gov/pubmed/28242719 (2017).

14. Shan, Y. *et al.* ATP-Dependent Persister Formation in Escherichia coli. *mBio* **8.** ISSN: 2150-7511 (Electronic). https://www.ncbi.nlm.nih.gov/pubmed/28174313 (2017).

15. Kudrin, P. *et al.* Subinhibitory Concentrations of Bacteriostatic Antibiotics Induce relA-Dependent and relA-Independent Tolerance to beta-Lactams. *Antimicrob Agents Chemother* **61.** ISSN: 1098-6596 (Electronic) 0066-4804 (Linking). https://www.ncbi.nlm.nih.gov/pubmed/28115345 (2017).

16. Dahl, J. U. *et al.* The anti-inflammatory drug mesalamine targets bacterial polyphosphate accumulation. *Nat Microbiol* **2,** 16267. ISSN: 2058-5276 (Electronic) 2058-5276 (Linking). https://www.ncbi.nlm.nih.gov/pubmed/28112760 (2017).

17. Lee, J. H., Kim, Y. G., Gwon, G., Wood, T. K. & Lee, J. Halogenated indoles eradicate bacterial persister cells and biofilms. *AMB Express* **6,** 123. ISSN: 2191-0855 (Print) 2191-0855 (Linking). https://www.ncbi.nlm.nih.gov/pubmed/27921270 (2016).

18. Molina-Quiroz, R. C., Lazinski, D. W., Camilli, A. & Levy, S. B. Transposon-Sequencing Analysis Unveils Novel Genes Involved in the Generation of Persister Cells in Uropathogenic Escherichia coli. *Antimicrob Agents Chemother* **60,** 6907–6910. ISSN: 1098-6596 (Electronic) 0066-4804 (Linking). https://www.ncbi.nlm.nih.gov/pubmed/27550350 (2016).

19. Orman, M. A. & Brynildsen, M. P. Persister formation in Escherichia coli can be inhibited by treatment with nitric oxide. *Free Radic Biol Med* **93,** 145–54. ISSN: 1873-4596 (Electronic) 0891-5849 (Linking). https://www.ncbi.nlm.nih.gov/pubmed/26849946 (2016).

20. Chowdhury, N., Kwan, B. W. & Wood, T. K. Persistence Increases in the Absence of the Alarmone Guanosine Tetraphosphate by Reducing Cell Growth. *Sci Rep* **6,** 20519. ISSN: 2045-2322 (Electronic) 2045-2322 (Linking). https://www.ncbi.nlm.nih.gov/pubmed/26837570 (2016).

21. Kim, J. S. *et al.* Fumarate-Mediated Persistence of Escherichia coli against Antibiotics. *Antimicrob Agents Chemother* **60,** 2232–40. ISSN: 1098-6596 (Electronic) 0066-4804 (Linking). https://www.ncbi.nlm.nih.gov/pubmed/26810657 (2016).

22. Amato, S. M. & Brynildsen, M. P. Persister Heterogeneity Arising from a Single Metabolic Stress. *Curr Biol* **25,** 2090–8. ISSN: 1879-0445 (Electronic) 0960-9822 (Linking). https://www.ncbi.nlm.nih.gov/pubmed/26255847 (2015).

23. Mordukhova, E. A. & Pan, J. G. Stabilization of homoserine-O-succinyltransferase (MetA) decreases the frequency of persisters in Escherichia coli under stressful conditions. *PLoS One* **9,** e110504. ISSN: 1932-6203 (Electronic) 1932-6203 (Linking). https://www.ncbi.nlm.nih.gov/pubmed/25329174 (2014).

24. Goneau, L. W. *et al.* Selective target inactivation rather than global metabolic dormancy causes antibiotic tolerance in uropathogens. *Antimicrob Agents Chemother* **58,** 2089–97. ISSN: 1098-6596 (Electronic) 0066-4804 (Linking). https://www.ncbi.nlm.nih.gov/pubmed/24449771 (2014).

25. Orman, M. A. & Brynildsen, M. P. Establishment of a method to rapidly assay bacterial persister metabolism. *Antimicrob Agents Chemother* **57,** 4398–409. ISSN: 1098-6596 (Electronic) 0066-4804 (Linking). https://www.ncbi.nlm.nih.gov/pubmed/23817376 (2013).

26. Orman, M. A. & Brynildsen, M. P. Dormancy is not necessary or sufficient for bacterial persistence. *Antimicrob Agents Chemother* **57,** 3230–9. ISSN: 1098-6596 (Electronic) 0066-4804 (Linking). https://www.ncbi.nlm.nih.gov/pubmed/23629720 (2013).

27. Leszczynska, D., Matuszewska, E., Kuczynska-Wisnik, D., Furmanek-Blaszk, B. & Laskowska, E. The formation of persister cells in stationary-phase cultures of Escherichia coli is associated with the aggregation of endogenous proteins. *PLoS One* **8,** e54737. ISSN: 1932-6203 (Electronic) 1932-6203 (Linking). https://www.ncbi.nlm.nih.gov/pubmed/23358116 (2013).

28. Wu, Y., Vulic, M., Keren, I. & Lewis, K. Role of oxidative stress in persister tolerance. *Antimicrob Agents Chemother* **56,** 4922–6. ISSN: 1098-6596 (Electronic) 0066-4804 (Linking). https://www.ncbi.nlm.nih.gov/pubmed/22777047 (2012).

29. Hong, S. H., Wang, X., O'Connor, H. F., Benedik, M. J. & Wood, T. K. Bacterial persistence increases as environmental fitness decreases. *Microb Biotechnol* **5,** 509–22. ISSN: 1751-7915 (Electronic) 1751-7915 (Linking). https://www.ncbi.nlm.nih.gov/pubmed/22221537 (2012).

30. Tashiro, Y. *et al.* RelE-mediated dormancy is enhanced at high cell density in Escherichia coli. *J Bacteriol* **194,** 1169–76. ISSN: 1098-5530 (Electronic) 0021-9193 (Linking). https://www.ncbi.nlm.nih.gov/pubmed/22210768 (2012).

31. Kim, J. S. *et al.* Bacterial persisters tolerate antibiotics by not producing hydroxyl radicals. *Biochem Biophys Res Commun* **413,** 105–10. ISSN: 1090-2104 (Electronic) 0006-291X (Linking). https://www.ncbi.nlm.nih.gov/pubmed/21872573 (2011).

32. Joers, A., Kaldalu, N. & Tenson, T. The frequency of persisters in Escherichia coli reflects the kinetics of awakening from dormancy. *J Bacteriol* **192,** 3379–84. ISSN: 1098-5530 (Electronic) 0021-9193 (Linking). https://www.ncbi.nlm.nih.gov/pubmed/20435730 (2010).

33. Ma, C. *et al.* Energy production genes sucB and ubiF are involved in persister survival and tolerance to multiple antibiotics and stresses in Escherichia coli. *FEMS Microbiol Lett* **303,** 33–40. ISSN: 1574-6968 (Electronic) 0378-1097 (Linking). https://www.ncbi.nlm.nih.gov/pubmed/20041955 (2010).

34. Kim, Y. & Wood, T. K. Toxins Hha and CspD and small RNA regulator Hfq are involved in persister cell formation through MqsR in Escherichia coli. *Biochem Biophys Res Commun* **391,** 209–13. ISSN: 1090-2104 (Electronic) 0006-291X (Linking). https://www.ncbi.nlm.nih.gov/pubmed/19909729 (2010).

35. Gefen, O., Gabay, C., Mumcuoglu, M., Engel, G. & Balaban, N. Q. Single-cell protein induction dynamics reveals a period of vulnerability to antibiotics in persister bacteria. *Proc Natl Acad Sci U S A* **105,** 6145–9. ISSN: 1091-6490 (Electronic) 0027-8424 (Linking). https://www.ncbi.nlm.nih.gov/pubmed/18427112 (2008).

36. Spoering, A. L., Vulic, M. & Lewis, K. GlpD and PlsB participate in persister cell formation in Escherichia coli. *J Bacteriol* **188,** 5136–44. ISSN: 0021-9193 (Print) 0021-9193 (Linking). https://www.ncbi.nlm.nih.gov/pubmed/16816185 (2006).

37. Balaban, N. Q., Merrin, J., Chait, R., Kowalik, L. & Leibler, S. Bacterial persistence as a phenotypic switch. *Science* **305,** 1622–5. ISSN: 1095-9203 (Electronic) 0036-8075 (Linking). https://www.ncbi.nlm.nih.gov/pubmed/15308767 (2004).

38. Keren, I., Kaldalu, N., Spoering, A., Wang, Y. & Lewis, K. Persister cells and tolerance to antimicrobials. *FEMS Microbiol Lett* **230,** 13–8. ISSN: 0378-1097 (Print) 0378-1097 (Linking). https://www.ncbi.nlm.nih.gov/pubmed/14734160 (2004).

39. Svenningsen, M. S., Veress, A., Harms, A., Mitarai, N. & Semsey, S. Birth and Resuscitation of (p)ppGpp Induced Antibiotic Tolerant Persister Cells. *Sci Rep* **9,** 6056. ISSN: 2045-2322 (Electronic) 2045-2322 (Linking). https://www.ncbi.nlm.nih.gov/pubmed/30988388 (2019).

40. Cahill, C. J., Pain, J. A. & Bailey, M. E. Gastrointestinal investigation of iron deficiency. *Br Med J (Clin Res Ed)* **293,** 269. ISSN: 0267-0623 (Print) 0267-0623 (Linking). https://www.ncbi.nlm.nih.gov/pubmed/3089484 (1986).

41. Chen, Z. *et al.* Hypoionic Shock Facilitates Aminoglycoside Killing of Both Nutrient Shift- and Starvation-Induced Bacterial Persister Cells by Rapidly Enhancing Aminoglycoside Uptake. *Front Microbiol* **10,** 2028. ISSN: 1664-302X (Print) 1664-302X (Linking). https://www.ncbi.nlm.nih.gov/pubmed/31551965 (2019).

42. Greenwood, D. & O'Grady, F. The effect of osmolality on the response of Escherichia coli and Proteus mirabilis to penicillins. *Br J Exp Pathol* **53,** 457–64. ISSN: 0007-1021 (Print).

43. Wolfson, J. S., Hooper, D. C., McHugh, G. L., Bozza, M. A. & Swartz, M. N. Mutants of Escherichia coli K-12 exhibiting reduced killing by both quinolone and beta-lactam antimicrobial agents. *Antimicrob. Agents Chemother.* **34,** 1938–1943 (1990).

44. Moyed, H. S. & Broderick, S. H. Molecular cloning and expression of hipA, a gene of Escherichia coli K-12 that affects frequency of persistence after inhibition of murein synthesis. *J. Bacteriol.* **166,** 399–403 (1986).

45. Moyed, H. S. & Bertrand, K. P. hipA, a newly recognized gene of Escherichia coli K-12 that affects frequency of persistence after inhibition of murein synthesis. *J. Bacteriol.* **155,** 768–775 (1983).

46. Greenwood, D. & O'Grady, F. Trimodal response of Escherichia coli and Proteus mirabilis to penicillins. *Nature* **228,** 457–8. ISSN: 0028-0836 (Print) 0028-0836 (Linking). https://www.ncbi.nlm.nih.gov/pubmed/4921466 (1970).

47. Pu, Y. *et al.* Enhanced Efflux Activity Facilitates Drug Tolerance in Dormant Bacterial Cells. *Mol Cell* **62,** 284–294. ISSN: 1097-4164 (Electronic) 1097-2765 (Linking). https://www.ncbi.nlm.nih.gov/pubmed/27105118 (2016).

48. Moker, N., Dean, C. R. & Tao, J. Pseudomonas aeruginosa increases formation of multidrug-tolerant persister cells in response to quorum-sensing signaling molecules. *J Bacteriol* **192,** 1946–55. ISSN: 1098-5530 (Electronic) 0021-9193 (Linking). https://www.ncbi.nlm.nih.gov/pubmed/20097861 (2010).

49. Tripathi, A., Dewan, P. C., Siddique, S. A. & Varadarajan, R. MazF-induced growth inhibition and persister generation in Escherichia coli. *J Biol Chem* **289,** 4191–205. ISSN: 1083-351X (Electronic) 0021-9258 (Linking). https://www.ncbi.nlm.nih.gov/pubmed/24375411 (2014).

50. Shah, D. *et al.* Persisters: a distinct physiological state of E. coli. *BMC Microbiol* **6,** 53. ISSN: 1471-2180 (Electronic) 1471-2180 (Linking). https://www.ncbi.nlm.nih.gov/pubmed/16768798 (2006).

51. Verstraeten, N. *et al.* Obg and Membrane Depolarization Are Part of a Microbial Bet-Hedging Strategy that Leads to Antibiotic Tolerance. *Mol Cell* **59,** 9–21. ISSN: 1097-4164 (Electronic) 1097-2765 (Linking). https://www.ncbi.nlm.nih.gov/pubmed/26051177 (2015).

52. Ghosh, A. *et al.* Contact-dependent growth inhibition induces high levels of antibiotic-tolerant persister cells in clonal bacterial populations. *EMBO J* **37.** ISSN: 1460-2075 (Electronic) 0261-4189 (Linking). https://www.ncbi.nlm.nih.gov/pubmed/29572241 (2018).

53. Choudhary, G. S. *et al.* Human Granulocyte Macrophage Colony-Stimulating Factor Enhances Antibiotic Susceptibility of Pseudomonas aeruginosa Persister Cells. *Sci Rep* **5,** 17315. ISSN: 2045-2322 (Electronic) 2045-2322 (Linking). https://www.ncbi.nlm.nih.gov/pubmed/26616387 (2015).

54. Kwan, B. W., Chowdhury, N. & Wood, T. K. Combatting bacterial infections by killing persister cells with mitomycin C. *Environ Microbiol* **17,** 4406–14. ISSN: 1462-2920 (Electronic) 1462-2912 (Linking). https://www.ncbi.nlm.nih.gov/pubmed/25858802 (2015).

55. Germain, E., Roghanian, M., Gerdes, K. & Maisonneuve, E. Stochastic induction of persister cells by HipA through (p)ppGpp-mediated activation of mRNA endonucleases. *Proc Natl Acad Sci U S A* **112,** 5171–6. ISSN: 1091-6490 (Electronic) 0027-8424 (Linking). https://www.ncbi.nlm.nih.gov/pubmed/25848049 (2015).

56. Dorr, T., Vulic, M. & Lewis, K. Ciprofloxacin causes persister formation by inducing the TisB toxin in Escherichia coli. *PLoS Biol* **8,** e1000317. ISSN: 1545-7885 (Electronic) 1544-9173 (Linking). https://www.ncbi.nlm.nih.gov/pubmed/20186264 (2010).

57. Dorr, T., Lewis, K. & Vulic, M. SOS response induces persistence to fluoroquinolones in Escherichia coli. *PLoS Genet* **5,** e1000760. ISSN: 1553-7404 (Electronic) 1553-7390 (Linking). https://www.ncbi.nlm.nih.gov/pubmed/20011100 (2009).

58. Brown, D. R. Nitrogen Starvation Induces Persister Cell Formation in Escherichia coli. *J Bacteriol* **201.** ISSN: 1098-5530 (Electronic) 0021-9193 (Linking). https://www.ncbi.nlm.nih.gov/pubmed/30420451 (2019).

59. Thao, S., Brandl, M. T. & Carter, M. Q. Enhanced formation of shiga toxin-producing Escherichia coli persister variants in environments relevant to leafy greens production. *Food Microbiol* **84,** 103241. ISSN: 1095-9998 (Electronic) 0740-0020 (Linking). https://www.ncbi.nlm.nih.gov/pubmed/31421768 (2019).

60. Edelmann, D. & Berghoff, B. A. Type I toxin-dependent generation of superoxide affects the persister life cycle of Escherichia coli. *Sci Rep* **9,** 14256. ISSN: 2045-2322 (Electronic) 2045-2322 (Linking). https://www.ncbi.nlm.nih.gov/pubmed/31582786 (2019).

61. Fleck, L. E. *et al.* A screen for and validation of prodrug antimicrobials. *Antimicrob Agents Chemother* **58,** 1410–9. ISSN: 1098-6596 (Electronic) 0066-4804 (Linking). https://www.ncbi.nlm.nih.gov/pubmed/24342644 (2014).

62. Meng, X., Nightingale, C. H., Sweeney, K. R. & Quintiliani, R. Loss of bactericidal activities of quinolones during the post-antibiotic effect induced by rifampicin. *J. Antimicrob. Chemother.* **33,** 721–728 (1994).

63. Mantilla-Calderon, D. & Hong, P. Y. Fate and Persistence of a Pathogenic NDM-1-Positive Escherichia coli Strain in Anaerobic and Aerobic Sludge Microcosms. *Appl Environ Microbiol* **83.** ISSN: 1098-5336 (Electronic) 0099-2240 (Linking). https://www.ncbi.nlm.nih.gov/pubmed/28411227 (2017).

64. Duan, X. *et al.* l-Serine potentiates fluoroquinolone activity against Escherichia coli by enhancing endogenous reactive oxygen species production. *J Antimicrob Chemother* **71,** 2192–9. ISSN: 1460-2091 (Electronic) 0305-7453 (Linking). https://www.ncbi.nlm.nih.gov/pubmed/27118777 (2016).

65. Carret, G., Flandrois, J. P. & Lobry, J. R. Biphasic kinetics of bacterial killing by quinolones. *J Antimicrob Chemother* **27,** 319–27. ISSN: 0305-7453 (Print) 0305-7453 (Linking). https://www.ncbi.nlm.nih.gov/pubmed/2037537 (1991).

66. Tkachenko, A. G., Kashevarova, N. M., Tyuleneva, E. A. & Shumkov, M. S. Stationary-phase genes upregulated by polyamines are responsible for the formation of Escherichia coli persister cells tolerant to netilmicin. *FEMS Microbiol Lett* **364.** ISSN: 1574-6968 (Electronic) 0378-1097 (Linking). https://www.ncbi.nlm.nih.gov/pubmed/28431088 (2017).

67. Tkachenko, A. G., Kashevarova, N. M., Karavaeva, E. A. & Shumkov, M. S. Putrescine controls the formation of Escherichia coli persister cells tolerant to aminoglycoside netilmicin. *FEMS Microbiol Lett* **361,** 25–33. ISSN: 1574-6968 (Electronic) 0378-1097 (Linking). https://www.ncbi.nlm.nih.gov/pubmed/25283595 (2014).

68. Kaspy, I. *et al.* HipA-mediated antibiotic persistence via phosphorylation of the glutamyl-tRNA-synthetase. *Nat Commun* **4,** 3001. ISSN: 2041-1723 (Electronic) 2041-1723 (Linking). https://www.ncbi.nlm.nih.gov/pubmed/24343429 (2013).

69. Mok, W. W., Orman, M. A. & Brynildsen, M. P. Impacts of global transcriptional regulators on persister metabolism. *Antimicrob Agents Chemother* **59,** 2713–9. ISSN: 1098-6596 (Electronic) 0066-4804 (Linking). https://www.ncbi.nlm.nih.gov/pubmed/25712354 (2015).

70. Kuczynska-Wisnik, D. *et al.* Lack of intracellular trehalose affects formation of Escherichia coli persister cells. *Microbiology* **161,** 786–96. ISSN: 1465-2080 (Electronic) 1350-0872 (Linking). https://www.ncbi.nlm.nih.gov/pubmed/25500492 (2015).

71. Amato, S. M., Orman, M. A. & Brynildsen, M. P. Metabolic control of persister formation in Escherichia coli. *Mol Cell* **50,** 475–87. ISSN: 1097-4164 (Electronic) 1097-2765 (Linking). https://www.ncbi.nlm.nih.gov/pubmed/23665232 (2013).

72. Hansen, S., Lewis, K. & Vulic, M. Role of global regulators and nucleotide metabolism in antibiotic tolerance in Escherichia coli. *Antimicrob Agents Chemother* **52,** 2718–26. ISSN: 1098-6596 (Electronic) 0066-4804 (Linking). https://www.ncbi.nlm.nih.gov/pubmed/18519731 (2008).

73. Greenwood, D. The activity of polymyxins against dense populations of Escherichia coli. *J Gen Microbiol* **91,** 110–8. ISSN: 0022-1287 (Print) 0022-1287 (Linking). https://www.ncbi.nlm.nih.gov/pubmed/172601 (1975).

74. Schmidt, N. W. *et al.* Engineering persister-specific antibiotics with synergistic antimicrobial functions. *ACS Nano* **8,** 8786–93. ISSN: 1936-086X (Electronic) 1936-0851 (Linking). https://www.ncbi.nlm.nih.gov/pubmed/25130648 (2014).

75. Singh, R., Ray, P., Das, A. & Sharma, M. Role of persisters and small-colony variants in antibiotic resistance of planktonic and biofilm-associated Staphylococcus aureus: an in vitro study. *J Med Microbiol* **58,** 1067–1073. ISSN: 0022-2615 (Print) 0022-2615 (Linking). https://www.ncbi.nlm.nih.gov/pubmed/19528167 (2009).

76. Bojer, M. S., Lindemose, S., Vestergaard, M. & Ingmer, H. Quorum Sensing-Regulated Phenol-Soluble Modulins Limit Persister Cell Populations in Staphylococcus aureus. *Front Microbiol* **9,** 255. ISSN: 1664-302X (Print) 1664-302X (Linking). https://www.ncbi.nlm.nih.gov/pubmed/29515541 (2018).

77. Kubistova, L., Dvoracek, L., Tkadlec, J., Melter, O. & Licha, I. Environmental Stress Affects the Formation of Staphylococcus aureus Persisters Tolerant to Antibiotics. *Microb Drug Resist* **24,** 547–555. ISSN: 1931-8448 (Electronic) 1076-6294 (Linking). https://www.ncbi.nlm.nih.gov/pubmed/28813617 (2018).

78. Conlon, B. P. *et al.* Persister formation in Staphylococcus aureus is associated with ATP depletion. *Nat Microbiol* **1,** 16051. ISSN: 2058-5276 (Electronic) 2058-5276 (Linking). https://www.ncbi.nlm.nih.gov/pubmed/27572649 (2016).

79. Mina, E. G. & Marques, C. N. Interaction of Staphylococcus aureus persister cells with the host when in a persister state and following awakening. *Sci Rep* **6,** 31342. ISSN: 2045-2322 (Electronic) 2045-2322 (Linking). https://www.ncbi.nlm.nih.gov/pubmed/27506163 (2016).

80. Sandvik, E. L., Fazen, C. H., Henry, T. C., Mok, W. W. & Brynildsen, M. P. Non-Monotonic Survival of Staphylococcus aureus with Respect to Ciprofloxacin Concentration Arises from Prophage-Dependent Killing of Persisters. *Pharmaceuticals (Basel)* **8,** 778–92. ISSN: 1424-8247 (Print) 1424-8247 (Linking). https://www.ncbi.nlm.nih.gov/pubmed/26593926 (2015).

81. Lechner, S., Lewis, K. & Bertram, R. Staphylococcus aureus persisters tolerant to bactericidal antibiotics. *J Mol Microbiol Biotechnol* **22,** 235–44. ISSN: 1660-2412 (Electronic) 1464-1801 (Linking). https://www.ncbi.nlm.nih.gov/pubmed/22986269 (2012).

82. Zalis, E. A. *et al.* Stochastic Variation in Expression of the Tricarboxylic Acid Cycle Produces Persister Cells. *mBio* **10.** ISSN: 2150-7511 (Electronic). https://www.ncbi.nlm.nih.gov/pubmed/31530676 (2019).

83. Sahukhal, G. S., Pandey, S. & Elasri, M. O. msaABCR operon is involved in persister cell formation in Staphylococcus aureus. *BMC Microbiol* **17,** 218. ISSN: 1471-2180 (Electronic) 1471-2180 (Linking). https://www.ncbi.nlm.nih.gov/pubmed/29166860 (2017).

84. Springer, M. T., Singh, V. K., Cheung, A. L., Donegan, N. P. & Chamberlain, N. R. Effect of clpP and clpC deletion on persister cell number in Staphylococcus aureus. *J Med Microbiol* **65,** 848–857. ISSN: 1473-5644 (Electronic) 0022-2615 (Linking). https://www.ncbi.nlm.nih.gov/pubmed/27375177 (2016).

85. Goncalves, F. D. & de Carvalho, C. C. Phenotypic Modifications in Staphylococcus aureus Cells Exposed to High Concentrations of Vancomycin and Teicoplanin. *Front Microbiol* **7,** 13. ISSN: 1664-302X (Print) 1664-302X (Linking). https://www.ncbi.nlm.nih.gov/pubmed/26834731 (2016).

86. Lechner, S., Patra, P., Klumpp, S. & Bertram, R. Interplay between population dynamics and drug tolerance of Staphylococcus aureus persister cells. *J Mol Microbiol Biotechnol* **22,** 381–91. ISSN: 1660-2412 (Electronic) 1464-1801 (Linking). https://www.ncbi.nlm.nih.gov/pubmed/23363876 (2012).

87. Aeschlimann, J. R., Hershberger, E. & Rybak, M. J. Analysis of vancomycin population susceptibility profiles, killing activity, and postantibiotic effect against vancomycin-intermediate Staphylococcus aureus. *Antimicrob Agents Chemother* **43,** 1914–8. ISSN: 0066-4804 (Print) 0066-4804 (Linking). https://www.ncbi.nlm.nih.gov/pubmed/10428912 (1999).

88. Soares, A. *et al.* Efficacy of a ciprofloxacin/amikacin combination against planktonic and biofilm cultures of susceptible and low-level resistant Pseudomonas aeruginosa. *J Antimicrob Chemother* **74,** 3252–3259. ISSN: 1460-2091 (Electronic) 0305-7453 (Linking). https://www.ncbi.nlm.nih.gov/pubmed/31424553 (2019).

89. Lee, S. & Bae, S. Molecular viability testing of viable but non-culturable bacteria induced by antibiotic exposure. *Microb Biotechnol* **11,** 1008–1016. ISSN: 1751-7915 (Electronic) 1751-7915 (Linking). https://www.ncbi.nlm.nih.gov/pubmed/29243404 (2018).

90. Manuel, J., Zhanel, G. G. & de Kievit, T. Cadaverine suppresses persistence to carboxypenicillins in Pseudomonas aeruginosa PAO1. *Antimicrob Agents Chemother* **54,** 5173–9. ISSN: 1098-6596 (Electronic) 0066-4804 (Linking). `https://www.ncbi.nlm.nih.gov/pubmed/20855735` (2010).

91. Spoering, A. L. & Lewis, K. Biofilms and planktonic cells of Pseudomonas aeruginosa have similar resistance to killing by antimicrobials. *J Bacteriol* **183,** 6746–51. ISSN: 0021-9193 (Print) 0021-9193 (Linking). `https://www.ncbi.nlm.nih.gov/pubmed/11698361` (2001).

92. Liebens, V. *et al.* Identification of 1-((2,4-Dichlorophenethyl)Amino)-3-Phenoxypropan-2-ol, a Novel Antibacterial Compound Active against Persisters of Pseudomonas aeruginosa. *Antimicrob Agents Chemother* **61.** ISSN: 1098-6596 (Electronic) 0066-4804 (Linking). `https://www.ncbi.nlm.nih.gov/pubmed/28630188` (2017).

93. Mlynarcik, P. & Kolar, M. Starvation- and antibiotics-induced formation of persister cells in Pseudomonas aeruginosa. *Biomed Pap Med Fac Univ Palacky Olomouc Czech Repub* **161,** 58–67. ISSN: 1213-8118 (Print) 1213-8118 (Linking). `https://www.ncbi.nlm.nih.gov/pubmed/27886280` (2017).

94. Cameron, D. R., Shan, Y., Zalis, E. A., Isabella, V. & Lewis, K. A Genetic Determinant of Persister Cell Formation in Bacterial Pathogens. *J Bacteriol* **200.** ISSN: 1098-5530 (Electronic) 0021-9193 (Linking). `https://www.ncbi.nlm.nih.gov/pubmed/29941425` (2018).

95. Niepa, T. H. *et al.* Sensitizing Pseudomonas aeruginosa to antibiotics by electrochemical disruption of membrane functions. *Biomaterials* **74,** 267–79. ISSN: 1878-5905 (Electronic) 0142-9612 (Linking). `https://www.ncbi.nlm.nih.gov/pubmed/26461119` (2016).

96. Marques, C. N., Morozov, A., Planzos, P. & Zelaya, H. M. The fatty acid signaling molecule cis-2-decenoic acid increases metabolic activity and reverts persister cells to an antimicrobial-susceptible state. *Appl Environ Microbiol* **80,** 6976–91. ISSN: 1098-5336 (Electronic) 0099-2240 (Linking). `https://www.ncbi.nlm.nih.gov/pubmed/25192989` (2014).

97. Niepa, T. H., Gilbert, J. L. & Ren, D. Controlling Pseudomonas aeruginosa persister cells by weak electrochemical currents and synergistic effects with tobramycin. *Biomaterials* **33,** 7356–65. ISSN: 1878-5905 (Electronic) 0142-9612 (Linking). `https://www.ncbi.nlm.nih.gov/pubmed/22840233` (2012).

98. Long, Y. *et al.* Identification of novel genes that promote persister formation by repressing transcription and cell division in Pseudomonas aeruginosa. *J Antimicrob Chemother* **74,** 2575–2587. ISSN: 1460-2091 (Electronic) 0305-7453 (Linking). `https://www.ncbi.nlm.nih.gov/pubmed/31139822` (2019).

99. Nierman, W. C., Yu, Y. & Losada, L. The In vitro Antibiotic Tolerant Persister Population in Burkholderia pseudomallei is Altered by Environmental Factors. *Front Microbiol* **6,** 1338. ISSN: 1664-302X (Print) 1664-302X (Linking). `https://www.ncbi.nlm.nih.gov/pubmed/26696964` (2015).

100. Allegretta, G. *et al.* In-depth Profiling of MvfR-Regulated Small Molecules in Pseudomonas aeruginosa after Quorum Sensing Inhibitor Treatment. *Front Microbiol* **8,** 924. ISSN: 1664-302X (Print) 1664-302X (Linking). `https://www.ncbi.nlm.nih.gov/pubmed/28596760` (2017).

101. Kaur, A., Sharma, P. & Capalash, N. Curcumin alleviates persistence of Acinetobacter baumannii against colistin. *Sci Rep* **8,** 11029. ISSN: 2045-2322 (Electronic) 2045-2322 (Linking). `https://www.ncbi.nlm.nih.gov/pubmed/30038318` (2018).

102. Chung, E. S. & Ko, K. S. Eradication of persister cells of Acinetobacter baumannii through combination of colistin and amikacin antibiotics. *J Antimicrob Chemother* **74,** 1277–1283. ISSN: 1460-2091 (Electronic) 0305-7453 (Linking). https://www.ncbi.nlm.nih.gov/pubmed/30759206 (2019).

103. Alkasir, R. *et al.* Characterization and Transcriptome Analysis of Acinetobacter baumannii Persister Cells. *Microb Drug Resist* **24,** 1466–1474. ISSN: 1931-8448 (Electronic) 1076-6294 (Linking). https://www.ncbi.nlm.nih.gov/pubmed/29902105 (2018).

104. Chung, E. S., Wi, Y. M. & Ko, K. S. Variation in formation of persister cells against colistin in Acinetobacter baumannii isolates and its relationship with treatment failure. *J Antimicrob Chemother* **72,** 2133–2135. ISSN: 1460-2091 (Electronic) 0305-7453 (Linking). https://www.ncbi.nlm.nih.gov/pubmed/28379382 (2017).

105. Donamore, B. K., Gallo, S. W., Abreu Ferreira, P. M., Sanchez Ferreira, C. A. & de Oliveira, S. D. Levels of persisters influenced by aeration in Acinetobacter calcoaceticus-baumannii. *Future Microbiol* **13,** 209–219. ISSN: 1746-0921 (Electronic) 1746-0913 (Linking). https://www.ncbi.nlm.nih.gov/pubmed/29302999 (2018).

106. Gallo, S. W., Donamore, B. K., Pagnussatti, V. E., Ferreira, C. A. & de Oliveira, S. D. Effects of meropenem exposure in persister cells of Acinetobacter calcoaceticus-baumannii. *Future Microbiol* **12,** 131–140. ISSN: 1746-0921 (Electronic) 1746-0913 (Linking). https://www.ncbi.nlm.nih.gov/pubmed/28111984 (2017).

107. Keren, I., Minami, S., Rubin, E. & Lewis, K. Characterization and transcriptome analysis of Mycobacterium tuberculosis persisters. *mBio* **2,** e00100–11. ISSN: 2150-7511 (Electronic). https://www.ncbi.nlm.nih.gov/pubmed/21673191 (2011).

108. Vilcheze, C. *et al.* Enhanced respiration prevents drug tolerance and drug resistance in Mycobacterium tuberculosis. *Proc Natl Acad Sci U S A* **114,** 4495–4500. ISSN: 1091-6490 (Electronic) 0027-8424 (Linking). https://www.ncbi.nlm.nih.gov/pubmed/28396391 (2017).

109. Choudhary, E., Sharma, R., Kumar, Y. & Agarwal, N. Conditional Silencing by CRISPRi Reveals the Role of DNA Gyrase in Formation of Drug-Tolerant Persister Population in Mycobacterium tuberculosis. *Front Cell Infect Microbiol* **9,** 70. ISSN: 2235-2988 (Electronic) 2235-2988 (Linking). https://www.ncbi.nlm.nih.gov/pubmed/30972304 (2019).

110. Torrey, H. L., Keren, I., Via, L. E., Lee, J. S. & Lewis, K. High Persister Mutants in Mycobacterium tuberculosis. *PLoS One* **11,** e0155127. ISSN: 1932-6203 (Electronic) 1932-6203 (Linking). https://www.ncbi.nlm.nih.gov/pubmed/27176494 (2016).

111. Hu, Y. *et al.* High-dose rifampicin kills persisters, shortens treatment duration, and reduces relapse rate in vitro and in vivo. *Front Microbiol* **6,** 641. ISSN: 1664-302X (Print) 1664-302X (Linking). https://www.ncbi.nlm.nih.gov/pubmed/26157437 (2015).

112. Guo, L. *et al.* Gas Plasma Pre-treatment Increases Antibiotic Sensitivity and Persister Eradication in Methicillin-Resistant Staphylococcus aureus. *Front Microbiol* **9,** 537. ISSN: 1664-302X (Print) 1664-302X (Linking). https://www.ncbi.nlm.nih.gov/pubmed/29628915 (2018).

113. Sieradzki, K., Leski, T., Dick, J., Borio, L. & Tomasz, A. Evolution of a vancomycin-intermediate Staphylococcus aureus strain in vivo: multiple changes in the antibiotic resistance phenotypes of a single lineage of methicillin-resistant S. aureus under the impact of antibiotics administered for chemotherapy. *J. Clin. Microbiol.* **41,** 1687–1693 (2003).

114. Walawalkar, Y. D., Vaidya, Y. & Nayak, V. Response of Salmonella Typhi to bile-generated oxidative stress: implication of quorum sensing and persister cell populations. *Pathog Dis* **74.** ISSN: 2049-632X (Electronic) 2049-632X (Linking). `https://www.ncbi.nlm.nih.gov/pubmed/27609462` (2016).

115. Silva-Herzog, E., McDonald, E. M., Crooks, A. L. & Detweiler, C. S. Physiologic Stresses Reveal a Salmonella Persister State and TA Family Toxins Modulate Tolerance to These Stresses. *PLoS One* **10,** e0141343. ISSN: 1932-6203 (Electronic) 1932-6203 (Linking). `https://www.ncbi.nlm.nih.gov/pubmed/26633172` (2015).

116. Rycroft, J. A. *et al.* Activity of acetyltransferase toxins involved in Salmonella persister formation during macrophage infection. *Nat Commun* **9,** 1993. ISSN: 2041-1723 (Electronic) 2041-1723 (Linking). `https://www.ncbi.nlm.nih.gov/pubmed/29777131` (2018).

117. Cheverton, A. M. *et al.* A Salmonella Toxin Promotes Persister Formation through Acetylation of tRNA. *Mol Cell* **63,** 86–96. ISSN: 1097-4164 (Electronic) 1097-2765 (Linking). `https://www.ncbi.nlm.nih.gov/pubmed/27264868` (2016).

118. Helaine, S. *et al.* Internalization of Salmonella by macrophages induces formation of nonreplicating persisters. *Science* **343,** 204–8. ISSN: 1095-9203 (Electronic) 0036-8075 (Linking). `https://www.ncbi.nlm.nih.gov/pubmed/24408438` (2014).

119. Drescher, S. P. M., Gallo, S. W., Ferreira, P. M. A., Ferreira, C. A. S. & Oliveira, S. D. Salmonella enterica persister cells form unstable small colony variants after in vitro exposure to ciprofloxacin. *Sci Rep* **9,** 7232. ISSN: 2045-2322 (Electronic) 2045-2322 (Linking). `https://www.ncbi.nlm.nih.gov/pubmed/31076596` (2019).

120. Braetz, S., Schwerk, P., Thompson, A., Tedin, K. & Fulde, M. The role of ATP pools in persister cell formation in (fluoro)quinolone-susceptible and -resistant strains of Salmonella enterica ser. Typhimurium. *Vet Microbiol* **210,** 116–123. ISSN: 1873-2542 (Electronic) 0378-1135 (Linking). `https://www.ncbi.nlm.nih.gov/pubmed/29103680` (2017).

121. Jaiswal, S. *et al.* The Hha-TomB Toxin-Antitoxin System Shows Conditional Toxicity and Promotes Persister Cell Formation by Inhibiting Apoptosis-Like Death in S. Typhimurium. *Sci Rep* **6,** 38204. ISSN: 2045-2322 (Electronic) 2045-2322 (Linking). `https://www.ncbi.nlm.nih.gov/pubmed/27910884` (2016).

122. Curtis, T. D., Gram, L. & Knudsen, G. M. The Small Colony Variant of Listeria monocytogenes Is More Tolerant to Antibiotics and Has Altered Survival in RAW 264.7 Murine Macrophages. *Front Microbiol* **7,** 1056. ISSN: 1664-302X (Print) 1664-302X (Linking). `https://www.ncbi.nlm.nih.gov/pubmed/27458449` (2016).

123. Knudsen, G. M., Ng, Y. & Gram, L. Survival of bactericidal antibiotic treatment by a persister subpopulation of Listeria monocytogenes. *Appl Environ Microbiol* **79,** 7390–7. ISSN: 1098-5336 (Electronic) 0099-2240 (Linking). `https://www.ncbi.nlm.nih.gov/pubmed/24056460` (2013).

124. Shapiro, J. A., Nguyen, V. L. & Chamberlain, N. R. Evidence for persisters in Staphylococcus epidermidis RP62a planktonic cultures and biofilms. *J Med Microbiol* **60,** 950–960. ISSN: 1473-5644 (Electronic) 0022-2615 (Linking). `https://www.ncbi.nlm.nih.gov/pubmed/21415203` (2011).

125. Leung, V. & Levesque, C. M. A stress-inducible quorum-sensing peptide mediates the formation of persister cells with noninherited multidrug tolerance. *J Bacteriol* **194,** 2265–74. ISSN: 1098-5530 (Electronic) 0021-9193 (Linking). `https://www.ncbi.nlm.nih.gov/pubmed/22366415` (2012).

126. Leung, V., Ajdic, D., Koyanagi, S. & Levesque, C. M. The formation of Streptococcus mutans persisters induced by the quorum-sensing peptide pheromone is affected by the LexA regulator. *J Bacteriol* **197,** 1083–94. ISSN: 1098-5530 (Electronic) 0021-9193 (Linking). https://www.ncbi.nlm.nih.gov/pubmed/25583974 (2015).

127. Willenborg, J., Willms, D., Bertram, R., Goethe, R. & Valentin-Weigand, P. Characterization of multi-drug tolerant persister cells in Streptococcus suis. *BMC Microbiol* **14,** 120. ISSN: 1471-2180 (Electronic) 1471-2180 (Linking). https://www.ncbi.nlm.nih.gov/pubmed/24885389 (2014).

128. Sharma, B., Brown, A. V., Matluck, N. E., Hu, L. T. & Lewis, K. Borrelia burgdorferi, the Causative Agent of Lyme Disease, Forms Drug-Tolerant Persister Cells. *Antimicrob Agents Chemother* **59,** 4616–24. ISSN: 1098-6596 (Electronic) 0066-4804 (Linking). https://www.ncbi.nlm.nih.gov/pubmed/26014929 (2015).

129. Caskey, J. R. & Embers, M. E. Persister Development by Borrelia burgdorferi Populations In Vitro. *Antimicrob Agents Chemother* **59,** 6288–95. ISSN: 1098-6596 (Electronic) 0066-4804 (Linking). https://www.ncbi.nlm.nih.gov/pubmed/26248368 (2015).

130. Feng, J. *et al.* Identification of novel activity against Borrelia burgdorferi persisters using an FDA approved drug library. *Emerg Microbes Infect* **3,** e49. ISSN: 2222-1751 (Print) 2222-1751 (Linking). https://www.ncbi.nlm.nih.gov/pubmed/26038747 (2014).

131. Ren, H. *et al.* Gradual increase in antibiotic concentration affects persistence of Klebsiella pneumoniae. *J Antimicrob Chemother* **70,** 3267–72. ISSN: 1460-2091 (Electronic) 0305-7453 (Linking). https://www.ncbi.nlm.nih.gov/pubmed/26311842 (2015).

132. Alvarez, R. *et al.* Effect of antibiotic treatment on the formation of non-spore Clostridium difficile persister-like cells. *J Antimicrob Chemother* **73,** 2396–2399. ISSN: 1460-2091 (Electronic) 0305-7453 (Linking). https://www.ncbi.nlm.nih.gov/pubmed/29757406 (2018).

133. Cha, R., Akins, R. L. & Rybak, M. J. Linezolid, levofloxacin, and vancomycin against vancomycin-tolerant and fluoroquinolone-resistant Streptococcus pneumoniae in an in vitro pharmacodynamic model. *Pharmacotherapy* **23,** 1531–7. ISSN: 0277-0008 (Print) 0277-0008 (Linking). https://www.ncbi.nlm.nih.gov/pubmed/14695032 (2003).

134. Liu, X. *et al.* Transcriptional Repressor PtvR Regulates Phenotypic Tolerance to Vancomycin in Streptococcus pneumoniae. *J Bacteriol* **199.** ISSN: 1098-5530 (Electronic) 0021-9193 (Linking). https://www.ncbi.nlm.nih.gov/pubmed/28484041 (2017).

135. Amraei, F., Narimisa, N., Sadeghi Kalani, B., Lohrasbi, V. & Masjedian Jazi, F. Persister cells formation and expression of type II Toxin-Antitoxin system genes in Brucella melitensis (16M) and Brucella abortus (B19). *Iran J Pathol* **15,** 127–133. ISSN: 1735-5303 (Print) 1735-5303 (Linking). https://www.ncbi.nlm.nih.gov/pubmed/32215028 (2020).

136. Butt, A. *et al.* Burkholderia pseudomallei kynB plays a role in AQ production, biofilm formation, bacterial swarming and persistence. *Res Microbiol* **167,** 159–67. ISSN: 1769-7123 (Electronic) 0923-2508 (Linking). https://www.ncbi.nlm.nih.gov/pubmed/26654915 (2016).

137. Tang, H. J. *et al.* In vitro and in vivo activities of newer fluoroquinolones against Vibrio vulnificus. *Antimicrob. Agents Chemother.* **46,** 3580–3584 (2002).

138. Van Tatenhove-Pel, R. J., Zwering, E., Solopova, A., Kuipers, O. P. & Bachmann, H. Ampicillin-treated Lactococcus lactis MG1363 populations contain persisters as well as viable but non-culturable cells. *Sci Rep* **9,** 9867. ISSN: 2045-2322 (Electronic) 2045-2322 (Linking). https://www.ncbi.nlm.nih.gov/pubmed/31285492 (2019).

139. Mukherjee, D., Zou, H., Liu, S., Beuerman, R. & Dick, T. Membrane-targeting AM-0016 kills mycobacterial persisters and shows low propensity for resistance development. *Future Microbiol* **11,** 643–50. ISSN: 1746-0921 (Electronic) 1746-0913 (Linking). https://www.ncbi.nlm.nih.gov/pubmed/27158932 (2016).
140. Sun, C. *et al.* MqsR/MqsA Toxin/Antitoxin System Regulates Persistence and Biofilm Formation in Pseudomonas putida KT2440. *Front Microbiol* **8,** 840. ISSN: 1664-302X (Print) 1664-302X (Linking). https://www.ncbi.nlm.nih.gov/pubmed/28536573 (2017).
141. John, J. F., Atkins, L. T., Maple, P. A. & Bratoeva, M. Activities of newer fluoroquinolones against Shigella sonnei. *Antimicrob. Agents Chemother.* **36,** 2346–2348 (1992).
142. Silva-Valenzuela, C. A. *et al.* Growth arrest and a persister state enable resistance to osmotic shock and facilitate dissemination of Vibrio cholerae. *ISME J* **11,** 2718–2728. ISSN: 1751-7370 (Electronic) 1751-7362 (Linking). https://www.ncbi.nlm.nih.gov/pubmed/28742070 (2017).
143. Van den Bergh, B. *et al.* Frequency of antibiotic application drives rapid evolutionary adaptation of Escherichia coli persistence. *Nat Microbiol* **1,** 16020. ISSN: 2058-5276 (Electronic) 2058-5276 (Linking). https://www.ncbi.nlm.nih.gov/pubmed/27572640 (2016).
144. Miyaue, S. *et al.* Bacterial Memory of Persisters: Bacterial Persister Cells Can Retain Their Phenotype for Days or Weeks After Withdrawal From Colony-Biofilm Culture. *Front Microbiol* **9,** 1396. ISSN: 1664-302X (Print) 1664-302X (Linking). https://www.ncbi.nlm.nih.gov/pubmed/29997606 (2018).
145. Cui, P. *et al.* Identification of Genes Involved in Bacteriostatic Antibiotic-Induced Persister Formation. *Front Microbiol* **9,** 413. ISSN: 1664-302X (Print) 1664-302X (Linking). https://www.ncbi.nlm.nih.gov/pubmed/29559967 (2018).
146. Bamford, R. A. *et al.* Investigating the physiology of viable but non-culturable bacteria by microfluidics and time-lapse microscopy. *BMC Biol* **15,** 121. ISSN: 1741-7007 (Electronic) 1741-7007 (Linking). https://www.ncbi.nlm.nih.gov/pubmed/29262826 (2017).
147. Luidalepp, H., Joers, A., Kaldalu, N. & Tenson, T. Age of inoculum strongly influences persister frequency and can mask effects of mutations implicated in altered persistence. *J Bacteriol* **193,** 3598–605. ISSN: 1098-5530 (Electronic) 0021-9193 (Linking). https://www.ncbi.nlm.nih.gov/pubmed/21602347 (2011).
148. Simsek, E. & Kim, M. Power-law tail in lag time distribution underlies bacterial persistence. *Proc Natl Acad Sci U S A* **116,** 17635–17640. ISSN: 1091-6490 (Electronic) 0027-8424 (Linking). https://www.ncbi.nlm.nih.gov/pubmed/31427535 (2019).
149. Aedo, S. J., Orman, M. A. & Brynildsen, M. P. Stationary phase persister formation in Escherichia coli can be suppressed by piperacillin and PBP3 inhibition. *BMC Microbiol* **19,** 140. ISSN: 1471-2180 (Electronic) 1471-2180 (Linking). https://www.ncbi.nlm.nih.gov/pubmed/31234796 (2019).
150. Bedenic, B. & Vranes, J. Post-exposure effects of cefepime and cefpirome on isogenic Escherichia coli hosts producing SHV-extended-spectrum beta-lactamases. *J Chemother* **15,** 342–9. ISSN: 1120-009X (Print) 1120-009X (Linking). https://www.ncbi.nlm.nih.gov/pubmed/12962362 (2003).
151. Chowdhury, N., Wood, T. L., Martinez-Vazquez, M., Garcia-Contreras, R. & Wood, T. K. DNA-crosslinker cisplatin eradicates bacterial persister cells. *Biotechnol Bioeng* **113,** 1984–92. ISSN: 1097-0290 (Electronic) 0006-3592 (Linking). https://www.ncbi.nlm.nih.gov/pubmed/26914280 (2016).

152. Windels, E. M. *et al.* Bacterial persistence promotes the evolution of antibiotic resistance by increasing survival and mutation rates. *ISME J* **13,** 1239–1251. ISSN: 1751-7370 (Electronic) 1751-7362 (Linking). `https://www.ncbi.nlm.nih.gov/pubmed/30647458` (2019).

153. Shan, Y., Lazinski, D., Rowe, S., Camilli, A. & Lewis, K. Genetic basis of persister tolerance to aminoglycosides in Escherichia coli. *mBio* **6.** ISSN: 2150-7511 (Electronic). `https://www.ncbi.nlm.nih.gov/pubmed/25852159` (2015).

154. Lebeaux, D. *et al.* pH-mediated potentiation of aminoglycosides kills bacterial persisters and eradicates in vivo biofilms. *J Infect Dis* **210,** 1357–66. ISSN: 1537-6613 (Electronic) 0022-1899 (Linking). `https://www.ncbi.nlm.nih.gov/pubmed/24837402` (2014).

155. Li, J., Ji, L., Shi, W., Xie, J. & Zhang, Y. Trans-translation mediates tolerance to multiple antibiotics and stresses in Escherichia coli. *J Antimicrob Chemother* **68,** 2477–81. ISSN: 1460-2091 (Electronic) 0305-7453 (Linking). `https://www.ncbi.nlm.nih.gov/pubmed/23812681` (2013).

156. Li, Y. *et al.* L-Tryptophan represses persister formation via inhibiting bacterial motility and promoting antibiotics absorption. *Future Microbiol* **14,** 757–771. ISSN: 1746-0921 (Electronic) 1746-0913 (Linking). `https://www.ncbi.nlm.nih.gov/pubmed/31271063` (2019).

157. Kawai, Y., Matsumoto, S., Ling, Y., Okuda, S. & Tsuneda, S. AldB controls persister formation in Escherichia coli depending on environmental stress. *Microbiol Immunol* **62,** 299–309. ISSN: 1348-0421 (Electronic) 0385-5600 (Linking). `https://www.ncbi.nlm.nih.gov/pubmed/29577369` (2018).

158. Matsumoto, S. *et al.* Unique transcriptional profile of native persisters in Escherichia coli. *J Biosci Bioeng* **125,** 15–22. ISSN: 1347-4421 (Electronic) 1347-4421 (Linking). `https://www.ncbi.nlm.nih.gov/pubmed/28821380` (2018).

159. VandenBerg, K. E., Ahn, S. & Visick, J. E. (p)ppGpp-Dependent Persisters Increase the Fitness of Escherichia coli Bacteria Deficient in Isoaspartyl Protein Repair. *Appl Environ Microbiol* **82,** 5444–54. ISSN: 1098-5336 (Electronic) 0099-2240 (Linking). `https://www.ncbi.nlm.nih.gov/pubmed/27371580` (2016).

160. Volzing, K. G. & Brynildsen, M. P. Stationary-Phase Persisters to Ofloxacin Sustain DNA Damage and Require Repair Systems Only during Recovery. *mBio* **6,** e00731–15. ISSN: 2150-7511 (Electronic). `https://www.ncbi.nlm.nih.gov/pubmed/26330511` (2015).

161. Goormaghtigh, F. & Van Melderen, L. Single-cell imaging and characterization of Escherichia coli persister cells to ofloxacin in exponential cultures. *Sci Adv* **5,** eaav9462. ISSN: 2375-2548 (Electronic) 2375-2548 (Linking). `https://www.ncbi.nlm.nih.gov/pubmed/31223653` (2019).

162. Wilmaerts, D. *et al.* HokB Monomerization and Membrane Repolarization Control Persister Awakening. *Mol Cell* **75,** 1031–1042 e4. ISSN: 1097-4164 (Electronic) 1097-2765 (Linking). `https://www.ncbi.nlm.nih.gov/pubmed/31327636` (2019).

163. Michiels, J. E., Van den Bergh, B., Verstraeten, N., Fauvart, M. & Michiels, J. In Vitro Emergence of High Persistence upon Periodic Aminoglycoside Challenge in the ESKAPE Pathogens. *Antimicrob Agents Chemother* **60,** 4630–7. ISSN: 1098-6596 (Electronic) 0066-4804 (Linking). `https://www.ncbi.nlm.nih.gov/pubmed/27185802` (2016).

164. Narayanaswamy, V. P. *et al.* Novel Glycopolymer Eradicates Antibiotic- and CCCP-Induced Persister Cells in Pseudomonas aeruginosa. *Front Microbiol* **9,** 1724. ISSN: 1664-302X (Print) 1664-302X (Linking). https://www.ncbi.nlm.nih.gov/pubmed/30123191 (2018).

165. Que, Y. A. *et al.* A quorum sensing small volatile molecule promotes antibiotic tolerance in bacteria. *PLoS One* **8,** e80140. ISSN: 1932-6203 (Electronic) 1932-6203 (Linking). https://www.ncbi.nlm.nih.gov/pubmed/24367477 (2013).

166. Van Laar, T. A. *et al.* Pseudomonas aeruginosa gshA Mutant Is Defective in Biofilm Formation, Swarming, and Pyocyanin Production. *mSphere* **3.** ISSN: 2379-5042 (Electronic) 2379-5042 (Linking). https://www.ncbi.nlm.nih.gov/pubmed/29669887 (2018).

167. Oppezzo, O. J. & Forte Giacobone, A. F. Lethal Effect of Photodynamic Treatment on Persister Bacteria. *Photochem Photobiol* **94,** 186–189. ISSN: 1751-1097 (Electronic) 0031-8655 (Linking). https://www.ncbi.nlm.nih.gov/pubmed/28881420 (2018).

168. Liebens, V. *et al.* Membrane localization and topology of the DnpA protein control fluoroquinolone tolerance in Pseudomonas aeruginosa. *FEMS Microbiol Lett* **363.** ISSN: 1574-6968 (Electronic) 0378-1097 (Linking). https://www.ncbi.nlm.nih.gov/pubmed/27481702 (2016).

169. Mulcahy, L. R., Burns, J. L., Lory, S. & Lewis, K. Emergence of Pseudomonas aeruginosa strains producing high levels of persister cells in patients with cystic fibrosis. *J Bacteriol* **192,** 6191–9. ISSN: 1098-5530 (Electronic) 0021-9193 (Linking). https://www.ncbi.nlm.nih.gov/pubmed/20935098 (2010).

170. Koeva, M. *et al.* An Antipersister Strategy for Treatment of Chronic Pseudomonas aeruginosa Infections. *Antimicrob Agents Chemother* **61.** ISSN: 1098-6596 (Electronic) 0066-4804 (Linking). https://www.ncbi.nlm.nih.gov/pubmed/28923873 (2017).

171. Wang, Y. *et al.* Inactivation of TCA cycle enhances Staphylococcus aureus persister cell formation in stationary phase. *Sci Rep* **8,** 10849. ISSN: 2045-2322 (Electronic) 2045-2322 (Linking). https://www.ncbi.nlm.nih.gov/pubmed/30022089 (2018).

172. Xu, T. *et al.* Absence of Protoheme IX Farnesyltransferase CtaB Causes Virulence Attenuation but Enhances Pigment Production and Persister Survival in MRSA. *Front Microbiol* **7,** 1625. ISSN: 1664-302X (Print) 1664-302X (Linking). https://www.ncbi.nlm.nih.gov/pubmed/27822202 (2016).

173. Vulin, C., Leimer, N., Huemer, M., Ackermann, M. & Zinkernagel, A. S. Prolonged bacterial lag time results in small colony variants that represent a sub-population of persisters. *Nat Commun* **9,** 4074. ISSN: 2041-1723 (Electronic) 2041-1723 (Linking). https://www.ncbi.nlm.nih.gov/pubmed/30287875 (2018).

174. Abouelhassan, Y., Basak, A., Yousaf, H. & Huigens R. W., r. Identification of N-Arylated NH125 Analogues as Rapid Eradicating Agents against MRSA Persister Cells and Potent Biofilm Killers of Gram-Positive Pathogens. *Chembiochem* **18,** 352–357. ISSN: 1439-7633 (Electronic) 1439-4227 (Linking). https://www.ncbi.nlm.nih.gov/pubmed/27925693 (2017).

175. Prax, M., Mechler, L., Weidenmaier, C. & Bertram, R. Glucose Augments Killing Efficiency of Daptomycin Challenged Staphylococcus aureus Persisters. *PLoS One* **11,** e0150907. ISSN: 1932-6203 (Electronic) 1932-6203 (Linking). https://www.ncbi.nlm.nih.gov/pubmed/26960193 (2016).

176. Garrison, A. T. *et al.* Halogenated Phenazines that Potently Eradicate Biofilms, MRSA Persister Cells in Non-Biofilm Cultures, and Mycobacterium tuberculosis. *Angew Chem Int Ed Engl* **54,** 14819–23. ISSN: 1521-3773 (Electronic) 1433-7851 (Linking). https://www.ncbi.nlm.nih.gov/pubmed/26480852 (2015).

177. Yee, R., Cui, P., Shi, W., Feng, J. & Zhang, Y. Genetic Screen Reveals the Role of Purine Metabolism in Staphylococcus aureus Persistence to Rifampicin. *Antibiotics (Basel)* **4,** 627–42. ISSN: 2079-6382 (Print) 2079-6382 (Linking). https://www.ncbi.nlm.nih.gov/pubmed/27025643 (2015).

178. Wang, W. *et al.* Transposon Mutagenesis Identifies Novel Genes Associated with Staphylococcus aureus Persister Formation. *Front Microbiol* **6,** 1437. ISSN: 1664-302X (Print) 1664-302X (Linking). https://www.ncbi.nlm.nih.gov/pubmed/26779120 (2015).

179. Xu, T. *et al.* The Agr Quorum Sensing System Represses Persister Formation through Regulation of Phenol Soluble Modulins in Staphylococcus aureus. *Front Microbiol* **8,** 2189. ISSN: 1664-302X (Print) 1664-302X (Linking). https://www.ncbi.nlm.nih.gov/pubmed/29163457 (2017).

180. Hu, Y., Coates, A. R. & Mitchison, D. A. Sterilizing activities of fluoroquinolones against rifampin-tolerant populations of Mycobacterium tuberculosis. *Antimicrob. Agents Chemother.* **47,** 653–657 (2003).

181. Curtis, T. D., Takeuchi, I., Gram, L. & Knudsen, G. M. The Influence of the Toxin/Antitoxin mazEF on Growth and Survival of Listeria monocytogenes under Stress. *Toxins (Basel)* **9.** ISSN: 2072-6651 (Electronic) 2072-6651 (Linking). https://www.ncbi.nlm.nih.gov/pubmed/28098783 (2017).

182. Nicol, M. *et al.* Anti-persister activity of squalamine against Acinetobacter baumannii. *Int J Antimicrob Agents* **53,** 337–342. ISSN: 1872-7913 (Electronic) 0924-8579 (Linking). https://www.ncbi.nlm.nih.gov/pubmed/30423343 (2019).

183. Kalita, S., Kandimalla, R., Bhowal, A. C., Kotoky, J. & Kundu, S. Functionalization of beta-lactam antibiotic on lysozyme capped gold nanoclusters retrogress MRSA and its persisters following awakening. *Sci Rep* **8,** 5778. ISSN: 2045-2322 (Electronic) 2045-2322 (Linking). https://www.ncbi.nlm.nih.gov/pubmed/29636496 (2018).

184. Kim, W. *et al.* A new class of synthetic retinoid antibiotics effective against bacterial persisters. *Nature* **556,** 103–107. ISSN: 1476-4687 (Electronic) 0028-0836 (Linking). https://www.ncbi.nlm.nih.gov/pubmed/29590091 (2018).

185. Kim, W. *et al.* Identification of an Antimicrobial Agent Effective against Methicillin-Resistant Staphylococcus aureus Persisters Using a Fluorescence-Based Screening Strategy. *PLoS One* **10,** e0127640. ISSN: 1932-6203 (Electronic) 1932-6203 (Linking). https://www.ncbi.nlm.nih.gov/pubmed/26039584 (2015).

186. Lee, J. S., Choi, J. Y., Chung, E. S., Peck, K. R. & Ko, K. S. Variation in the formation of persister cells against meropenem in Klebsiella pneumoniae bacteremia and analysis of its clinical features. *Diagn Microbiol Infect Dis* **95,** 114853. ISSN: 1879-0070 (Electronic) 0732-8893 (Linking). https://www.ncbi.nlm.nih.gov/pubmed/31353067 (2019).

187. Duan, X. *et al.* Mycobacterium Lysine epsilon-aminotransferase is a novel alarmone metabolism related persister gene via dysregulating the intracellular amino acid level. *Sci Rep* **6,** 19695. ISSN: 2045-2322 (Electronic) 2045-2322 (Linking). https://www.ncbi.nlm.nih.gov/pubmed/26806099 (2016).
